# Hippocampal interleukin-33 mediates neuroinflammation-induced cognitive impairments

**DOI:** 10.1186/s12974-020-01939-6

**Published:** 2020-09-11

**Authors:** Flora Reverchon, Vidian de Concini, Vanessa Larrigaldie, Sulayman Benmerzoug, Sylvain Briault, Dieudonnée Togbé, Bernhard Ryffel, Valérie F. J. Quesniaux, Arnaud Menuet

**Affiliations:** 1 UMR7355, Experimental and Molecular Immunology and Neurogenetics, CNRS and University of Orléans, 3B rue de la Ferollerie, 45071 Orléans, France; 2 Current address: Center for Molecular Biophysics, CNRS UPR4301, 45071 Orléans, France; 3 Current address:Department of Urology, Urology Research Unit, CHUV, Lausanne, Switzerland; 4 Department of Genetics, Regional Hospital, Orléans, France; 5 Artimmune SAS, 13 Avenue Buffon, 45071 Orléans-Cedex 2, France

**Keywords:** Interleukin-33, Interleukin-1, Microglia, Memory

## Abstract

**Background:**

Interleukin (IL)-33 is expressed in a healthy brain and plays a pivotal role in several neuropathologies, as protective or contributing to the development of cerebral diseases associated with cognitive impairments. However, the role of IL-33 in the brain is poorly understood, raising the question of its involvement in immunoregulatory mechanisms.

**Methods:**

We administered recombinant IL-33 (rmIL-33) by intra-hippocampal injection to C57BL/6 J (WT) and IL-1αβ deficient mice. Chronic minocycline administration was performed and cognitive functions were examined trough spatial habituation test. Hippocampal inflammatory responses were investigated by RT-qPCR. The microglia activation was assessed using immunohistological staining and fluorescence-activated cell sorting (FACS).

**Results:**

We showed that IL-33 administration in mice led to a spatial memory performance defect associated with an increase of inflammatory markers in the hippocampus while minocycline administration limited the inflammatory response. Quantitative assessment of glial cell activation in situ demonstrated an increase of proximal intersections per radius in each part of the hippocampus. Moreover, rmIL-33 significantly promoted the outgrowth of microglial processes. Fluorescence-activated cell sorting analysis on isolated microglia, revealed overexpression of IL-1β, 48 h post-rmIL-33 administration. This microglial reactivity was closely related to the onset of cognitive disturbance. Finally, we demonstrated that IL-1αβ deficient mice were resistant to cognitive disorders after intra-hippocampal IL-33 injection.

**Conclusion:**

Thus, hippocampal IL-33 induced an inflammatory state, including IL-1β overexpression by microglia cells, being causative of the cognitive impairment. These results highlight the pathological role for IL-33 in the central nervous system, independently of a specific neuropathological model.

## Introduction

IL-33 is a member of the interleukin-1 (IL-1) cytokine family that plays important roles in various disorders including allergy, autoimmune, or cardiovascular diseases through its receptor ST2 and co-receptor IL-1 accessory protein (IL-1RAcP) [1]. Recently, IL-33 has also been involved in the pathogenesis of central nervous system (CNS) diseases such as neurodegenerative diseases, stroke, or infectious diseases. Broadly and highly expressed in the CNS in physiological conditions, IL-33 is described as a key regulator of neuroinflammation [2–4].

In experimental autoimmune encephalomyelitis (EAE), a model of multiple sclerosis disease (MS), a systemic administration of recombinant IL-33, from the day of immunization until day 18, induces a protective effect [5]. However, the intraperitoneal administration of anti-IL-33 neutralizing antibodies also delayed the onset and the severity of EAE [6]. These apparently opposite findings highlight the dual function of IL-33. Moreover, this dual function of IL-33 has also been observed in Alzheimer’s disease (AD). IL-33 is highly expressed in the vicinity of amyloid plaques and in glial cells in brain sections from AD patients suggesting that a prolonged IL-33 production may induce inflammatory molecule release and contribute to the AD pathogenesis with neuronal damage [7]. However, more recently, Saresella et al. [8] demonstrated a decrease of IL-33 in the serum of AD patients as compared with healthy controls. These clinical data highlight a complex pro- and anti-inflammatory properties of IL-33 in AD patients acting both at the central and systemic level. IL-33 dual functions have also been observed in CNS infectious diseases. We previously reported the essential role of the IL-33 receptor ST2 in the pathogenesis of experimental cerebral malaria (ECM) caused by *Plasmodium berghei* Anka (*Pb*A)-infection in mice. We showed that ST2-deficient mice were resistant to *Pb*A-induced neuropathology [9] and demonstrated a deleterious role of CNS endogenous IL-33 in the neuropathogenesis associated with cognitive disorders [10]. Surprisingly, IL-33 deficient mice were not resistant to ECM [11] and IL-33 systemic administration improved antimalarial drug treatment of ECM via Treg cells [12, 13]. Thus, IL-33 has dual effects on infection, inflammation, and diseases of the CNS [1] raising the question of the cellular and immunomodulators involved.

Immunohistological analyses and IL-33/citrine reporter mice showed that astrocytes [14, 15] and oligodendrocytes [10, 16] are the main cellular sources of IL-33 within the CNS. Moreover, ST2 receptor is overexpressed by astrocytes and microglial cells under pathophysiological conditions [14]. Microglia could be the first glial cells to respond to IL-33 stimulation through the ST2/IL-1RAcP receptor complex [15]. We previously showed a deleterious effect of CNS endogenous IL-33 through the activation of microglia leading to IL-1β release in ECM [10]. IL-33 is not only involved [15, 16] but essential for the microglial activation [17]. Given the importance of microglia in the neurotoxic or neuroprotective inflammatory responses, CNS IL-33 may be a key factor in the neuroinflammatory processes and associated with cognitive impairments.

In this study, we show that recombinant mouse IL-33 administration in the hippocampus led to microglial cell activation and increased IL-1 production associated with cognitive disturbance.

## Materials and methods

### Mice and ethics statement

C57BL/6 J (wild-type; WT) male mice under specific pathogen-free (SPF) condition at 8 weeks of age were purchased from Janvier Labs (Le Genest Saint Isle, France). Mice deficient for both IL-1α and IL-1β were bred in the Transgenose Institute animal facility (CNRS UPS44, Orleans, France). They were issued from an intercross between IL-1α ΚΟ and IL-1β ΚΟ mice [18]. As they were backcrossed 10-fold on C57BL/6 J background, C57BL/6 J control was used. Mice were housed at four per propylene cage with woodchip bedding, and kept under controlled conditions of temperature (20–22 °C), humidity (50%), and bright cycle (12/12-h dark/light), with free access to chow pellets and water. The animals were previously habituated to our animal facility at 4 weeks and used in experimental settings at 8 weeks of age. All animal experimental protocols complied with the French ethical and animal experiments regulations (see Charte Nationale, Code Rural R 214–122, 214–124 and European Union Directive 86/609/EEC) and were approved by the “Ethics Committee for Animal Experimentation of CNRS Campus Orleans” (CCO), registered (N°3) by the French National Committee of Ethical Reflexion for Animal Experimentation, under N° CLE CCO 2015-1084 and by the French “Ministère de l’enseignement supérieur, de la recherche et de l’innovation”, under number APAFIS #19264.

### Intrahippocampal microinjection

Mice divided into 4 groups, received intrahippocampal injections of either vehicle PBS containing 0.1% BSA as a carrier (PBS-BSA) or recombinant mouse (rm) IL-33 protein (R&D Systems, Abingdon, UK; 200 ng/μl in PBS-BSA), in the absence or in the presence of minocycline hydrochloride (MP Biomedicals, Illkirch, France) was administered daily (i.p, 50 mg/kg in NaCl 0.9%) during 10 days, including 7 days before surgery and 3 days post-surgery. Before intrahippocampal injections, mice anesthetized with ketamine/xylazine (100 μL/10 g i.p. of 29.4 mg/mL ketamine plus 3.05 mg/mL xylazine) were secured in the stereotaxic apparatus (KOPF instruments, Lidingö, Sweden). Burr holes were drilled bilaterally in the skull above the hippocampus at 2.0 mm posterior to bregma, and ±1.8 mm lateral to bregma. Then, mice received bilateral intrahippocampal injection of rmIL-33 protein at 400 ng in a total volume of 2 μL of PBS-BSA by side. Control animals received PBS-BSA vehicle. A 10-μL Hamilton syringe (Hamilton, Reno, NV, USA) controlled by a Stereotaxic Injector (KD Scientific, Holliston, USA) was used to inject the solution at a rate of 0.25 μL/min in the hippocampus at −1.80 mm to Bregma. After the surgery and to facilitate recovery, each mouse was placed alone per cage until the end of the experiments. Groups of sham animals were subjected to a similar hippocampal surgery, without PBS-BSA or rmIL-33 injection with or without minocycline pretreatment.

### Spatial habituation test

Spatial habituation to a novel environment is commonly used for the exploration of non-associative learning and memory processes linked to hippocampal structures [19–21]. As previously described [22], to explore the learning component, 1 day after the surgical intervention, the animal was allowed to explore an open field (OF) (40 cm × 40 cm) for 10 min (trial session). After 24 h, the mouse was re-exposed for 10 min to the same OF (test session). During each session, the exploratory measures were quantified using the Ethovision tracking system (version 10, Noldus Technology, Wageningen, Netherlands). Locomotor activity was indexed by the distance traveled in the entire open-field arena. To explore intrasession habituation during the trial session, the distance traveled between the first and the last minute was compared. The intersession habituation was assessed by comparing the full distance traveled during both sessions. All sessions were performed at 10 lux to limit the anxiogenic component of the novel environment.

### Real-time quantitative polymerase chain reaction (RT-qPCR)

At the indicated time, total RNA from the hippocampus was isolated using TRI-Reagent (Sigma-Aldrich, Saint-Quentin Fallavier, France) as previously described [10] and reverse transcripted (Superscript III reverse transcriptase, Invitrogen, Carlsbad, CA). Quantitative real-time PCR reactions were performed using GoTaq qPCR-Master Mix (Promega, Charbonnières-les-Bains, France) and primers for *Nos2*, *Il1b, Tnfa*, *Ifng*, *Arg1, Chil3, Il10, and Igf1* (Qiagen, Hilden, Germany). After normalization using *18S*-RNA expression as a housekeeping gene, raw data were analyzed by the 2^ΔΔCt^ method [23].

### Tissue preparation and immunofluorescence

For immunostaining, mice were deeply anesthetized and transcardially perfused with ice-cold PBS followed by 4% paraformaldehyde (PFA). The brains were collected, post-fixed for 48 h in 4% PFA, and cryo-protected in a 30% sucrose solution for 1 week. Then, 14 μm brain cryo-sections mounted onto glass slides were incubated in citrate buffer (pH = 6) at 80 °C for 30 min, followed by incubation with blocking solution (TBS 1X; 1% BSA; 10% FCS; 0.3% Triton; 1% NaN3) during 45 min in a wet chamber at room temperature. After incubation overnight at 4 °C with anti-Iba-1 antibody (Abcam, Cambridge, England, ab5076; 1:500), the sections were washed in TBS and incubated with Alexa 488 secondary antibody (Abcam, ab150129, 1:1000) for 1 h. The slides were rinsed, then counter-stained with DAPI for 10 min, mounted with Fluoromount-G (SouthernBiotech, Birmingham, England), and dried before observation using ZEISS AXIOVERT 200 M/Apotome microscope (Zeiss, Oberkochen, Germany). Serial sections were collected at ×20 magnification to reconstruct each whole-hippocampal image software (ZEN2.1, Zeiss). The images were collected as Z-series of 18 optical slices to obtain a sufficient resolution to perform the morphological analysis of microglial cells. For each mouse, 3 representative stacks of images of the hippocampus were recorded. Positive cells for Iba-1 were counted (50–100 cells) and their morphology analyzed in each area e.g. the cornu ammonis (CA)1/CA2, CA3 and the dentate gyrus (DG). Image analysis and processing were performed with the software Image J -Fiji [24] using the “concentric circles” plugin. For the Sholl analysis, the intersection number per radian was defined each 5 μm from the center of each cell (*n* = 3 mice per treatment with 50-100 microglia analyzed per mouse). This analysis was performed by a blinded experimenter.

### Fluorescence-activated cell sorting

The hippocampus of 3 mice perfused with phosphate-buffer saline (PBS) was pooled and the cellular suspensions were prepared using the Neural Tissue Dissociation Kit (Miltenyi Biotec, Paris France), according to the manufacturer’s instructions. Cells were stained with extracellular conjugated antibodies: Fixable Viability Dye (eBiosciences™, 65-0865-14, 1/800), anti-CD45 V450 (BD Horizon™, 560501, 1:100), anti-CD11b PerCP/Cy5 (BD Pharmingen™, 560993, 1:100) and blocked with non-conjugated anti-CD16/32 (BD Pharmingen™, 553142, 1:100) for 20 min at 4 °C. Then, the cells were washed before fixation. Intracellular IL-1β pro-form stained with PE-conjugated specific antibody (eBioscience™, 12-7114-80, 1:20) was visualized after cell permeabilization for 20 min at 4 °C with Cytofix/Cytoperm Plus Kit (BD Biosciences, Paris, France). This antibody recognizes only the pro-form of mouse IL-1β and does not detect the cleaved and secreted mature IL-1β form. Cells were then washed and re-suspended in lysing solution (BD FACS™ Lysing Solution) before the acquisition. Data were acquired with a flow cytometer (BD FACSCanto II) and analyzed with FlowJo v7.6.5 software (Tree Star, Ashland, OR). Very low SSC and very low FSC were excluded to strictly define the populations of interest. IL-1β pro-form staining was measured using geometric mean fluorescence intensity (GMFI). For the analysis, live single cells were pre-gated. Then, CD11b^+^/CD45^low^ cells were gated as microglia, while CD11b^+^/CD45^high^ cells or CD11b^−^/CD45^high^ cells were gated as infiltrating macrophage or lymphocyte cells, respectively. FMO controls were also included to define populations of Fixable Viability Dye cells and CD45, CD11b, and IL-1β-expressing cells.

### Statistical

Statistical significance was determined with GraphPad Prism v6 (GraphPad Software, La Jolla, CA). Standard errors of the mean are reported as SEM. To analyze non-parametric data, Mann-Whitney test for 2 series was used or Kruskal-Wallis followed by Dunn’s multiple comparison for more series. *P* values ≤ 0.05 were considered statistically significant.

## Results

### Local hippocampal rmIL-33 injection impairs long-term memory

**Fig. 1:**
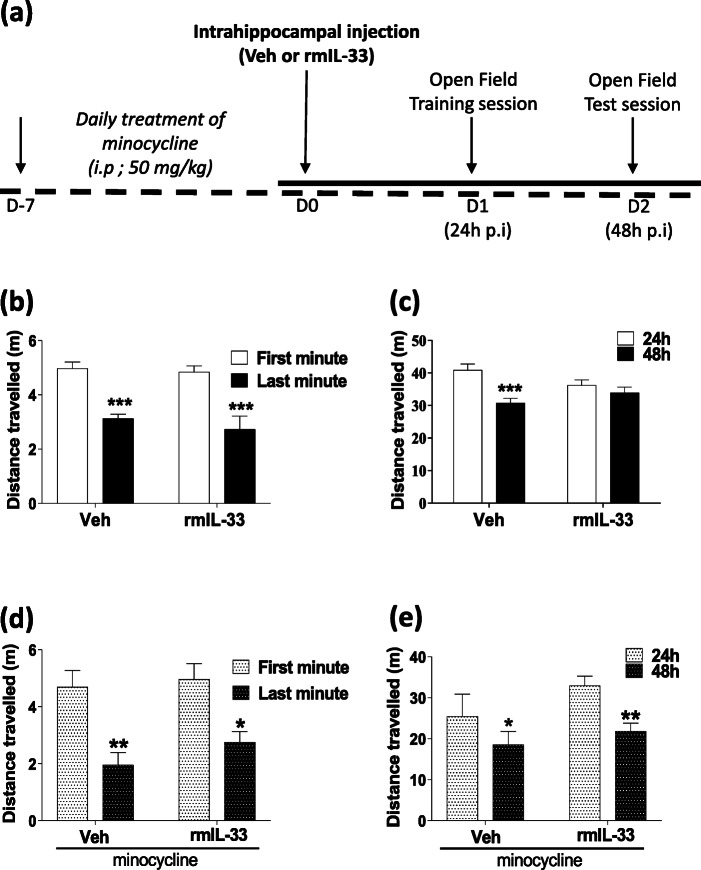
Effect of hippocampal exogenous IL-33 associated with minocycline pre-treatment on spatial memory and habituation. **a** Experimental design. Mice were intrahippocampally injected with rmIL-33 or vehicle solution (PBS-BSA 0.1%). The cognitive behavior was tested on day 1 by a first open-field session, followed by a second session on day 2. The short term habituation was analyzed during the first session on day 1. **b** and **d** The distance traveled between the first and the last minute in the open-field were compared. **c** and **e** The long-term habituation was accessed by comparing the distance traveled between the first session (24 h) and the last session (48 h). Two cohorts have been studied: (**b** and **c**) vehicle or rmIL-33 treated without minocycline, (**d** and **e**) vehicle or rmIL-33 treated with minocycline. Under our experimental conditions, IL-33 impaired the intersession habituation (**c**). This effect on spatial memory retrieval was prevented by minocycline administration (**e**). Values are mean ± SEM, *n* = 8–15 per group corresponding to a pool of 2 independent experiments. Two-way ANOVA followed by a Sidak post hoc test was used to analyze the distance traveled among groups. *P ≤ 0.05, **P ≤ 0.01, ***P ≤ 0.001

We previously proposed a role for IL-33/ST2 signaling pathway in the hippocampus in the cognitive impairments after *Pb*A-infection, especially on the memory process [10]. In this respect, we asked whether CNS IL-33 overexpression, mimicked here by an exogenous rmIL-33 administration locally in the hippocampus, could influence cognitive functions. After bilateral intrahippocampal injections of rmIL-33 or vehicle, non-associative learning and memory retrieval processes were explored by spatial habituation test in an open-field apparatus as described in Fig. 1a. The time spent in the central square was similar in all tested groups 24 h post-surgery (Addition file 1), suggesting an absence of specific anxiogenic response. The total distance traveled 24 h post-surgery decreased from 1–10 min during the first session in a novel environment for both vehicle- and rmIL-33-treated mice (Fig. 1b), corresponding to appropriate habituation to spatial novelty. The total distance traveled during the test session at 48 h was significantly reduced in the vehicle group, as compared with the training session at 24 h, indicating a normal ability to retrieve the previous exploratory information from memory processes, e.g., a proper long-term habituation process (Fig. 1c). In contrast, rmIL-33-treated mice showed no reduction of distance traveled at 48 h, as compared with the 24h training session, indicative of a disturbed long-term habituation process (Fig. 1c). These findings showed that rmIL-33 hippocampal administration impaired spatial memory retrieval processes.

### Minocycline prevents the long-term memory impairment induced by rmIL-33

IL-33 is considered as an immunomodulator of various neuropathologies [2]. To investigate the impact of the immune response on rmIL-33-induced cognitive impairment, minocycline which is an anti-inflammatory antibiotic able to cross the blood-brain barrier [25] was used. Minocycline pre-treatment was administrated daily, starting 7 days prior to vehicle and rmIL-33 intra-hippocampal administration. In our experimental conditions (Fig. 1a), minocycline treatment did not affect anxiogenic response to a novel environment (Addition file 1). In addition, habituation to spatial novelty in vehicle or rmIL-33-treated animals during the trial session was also conserved (Fig. 1d). In contrast, the impairment of long-term habituation previously observed after rmIL-33 administration (Fig. 1c) was absent in minocycline-treated animals (Fig. 1e). Indeed, mice receiving minocycline treatment showed a decrease in distance traveled at 48 h compared with the distance traveled at 24 h even after rm-IL-33 administration. These data thus suggest that minocycline treatment prevents the deleterious effect of rmIL-33 administration on spatial memory retrieval. These data demonstrate that minocycline should prevent the deleterious effect of rmIL-33 administration on spatial memory retrieval.

### IL-33 drives inflammatory response in the hippocampus

**Fig. 2:**
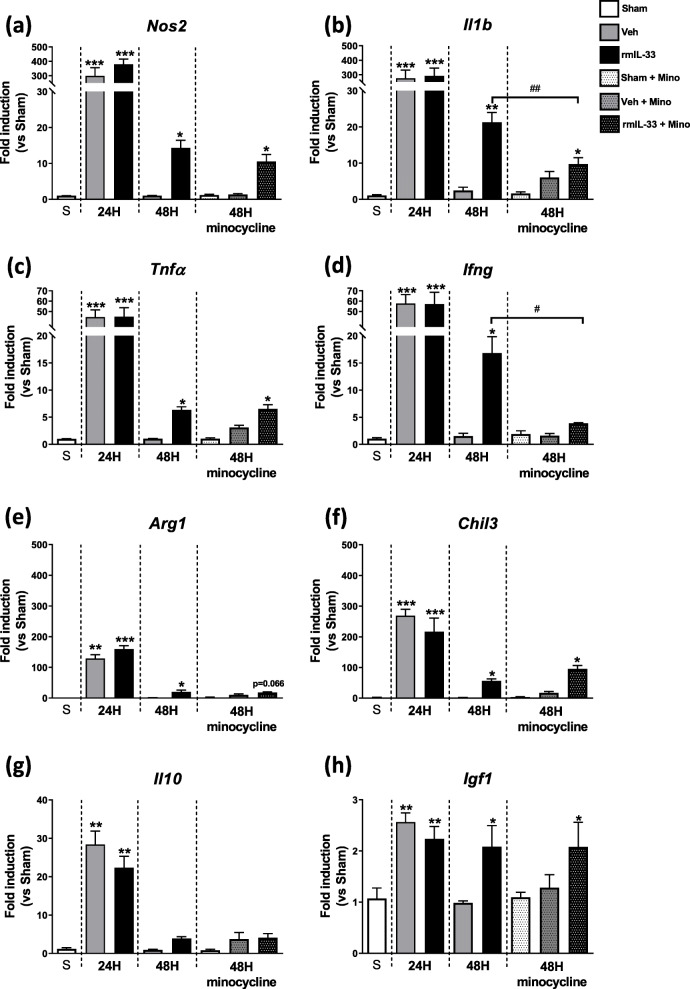
mRNA expression of inflammatory markers in hippocampi of IL-33-injected mice. mRNA expression of pro-inflammatory markers (*Nos2* in **a**, *IL1b* in **b**, *Tnfa* in **c** and *Ifng* in **d**) or anti-inflammatory markers (*Arg1* in **e**, *Chil3* in **f**, *IL10* in **g**, and *Igf1* in **h**) were quantified in hippocampi at 24 h and 48 h post-surgery by RT-qPCR normalized against *18S* RNA. Relative fold change in vehicle group (grey bar) and in rmIL-33 group (black bar) were quantified versus sham group (S; white bar). Minocycline-treated mice were also analyzed at 48 h post-surgery (dotted bar). IL-33 injection delayed the resolution of inflammation highlighted by an increase of inflammatory markers at 48 h administration. A partial reduction of this effect was observed under minocycline exposure, especially for *IL-1b* and *Ifng* mRNA. Data are represented as mean ± SEM (*n* = 4–6). Statistical comparisons were made using Kruskal-Wallis followed by Dunn’s multiple comparison test for each group vs. Sham. *P ≤ 0.05, **P ≤ 0.01, ***P ≤ 0.001. In addition, a comparison between rmIL-33 (48 h) and rmIL-33 + mino (48 h) groups was performed using the Mann-Whitney test. #*P* ≤ 0.05, ##*P* ≤ 0.01

We next asked whether the effect of minocycline on restoring rmIL-33-induced cognitive impairment may be associated with its reduction of a neuroinflammatory response [25]. We evaluated the time course of neuroinflammatory processes in the hippocampus 24 h and 48 h after a single injection of rmIL-33 alone or in the presence of minocycline pre-treatment in the hippocampus (Fig. 2). The slight increase in pro-inflammatory markers expression seen at 24 h post-injection in terms of *Nos2*, *Il1b*, *Tnfa*, *Ifng* (Fig. 2a to d), as well as anti-inflammatory markers *Arg1*, *Chil3*, *Il10* and *Igf1* (Fig. 2e to h) was observed both in vehicle- and rmIL-33-treated mice, as compared with the sham group, suggesting an inflammatory response to the microinjection itself. This inflammatory response was resolved at 48 h in vehicle-treated control animals, returning to the level of the sham group. However, at 48 h, a time point corresponding to the cognitive impairment, rmIL-33-treated mice showed high levels of hippocampal expression of inflammatory markers, as compared with the vehicle group. Thus, rmIL-33 administration delayed the resolution of inflammation. Interestingly, minocycline treatment reduced the expression of *Il1b* and *Ifng* observed 48 h after the rmIL-33 administration (Fig. 2b, d) while it had no effect on the other parameters studied. Thus, minocycline treatment partially reduces the deleterious effects of rmIL-33 on the resolution of inflammation by limiting the overexpression of *Il1b* and *Ifng*. We must notice that *Il1a* expression analysis showed a similar response to *Il1b* but fold inductions were widely reduced (Addition file 2).

### Intrahippocampal administration of rmIL-33 exacerbates microglial activation

**Fig. 3:**
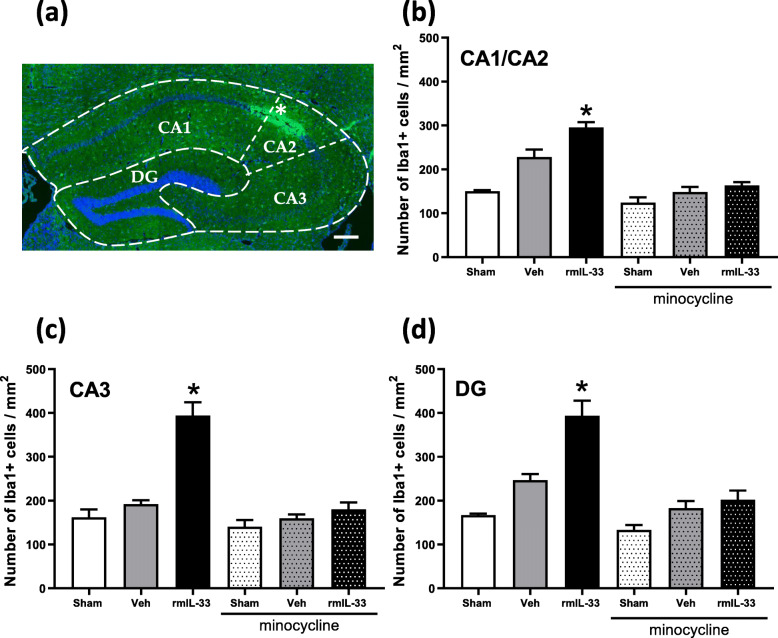
Effects of rmIL-33 treatment on the number of microglial cells in the hippocampal areas. Mice were intrahippocampally injected with rmIL-33 or vehicle solution (PBS-BSA 0.1%) with or without minocycline pretreatment as in Fig. 1a and brain sections analyzed at 48 h. **a** Representative immunohistochemical staining (Dapi in blue and Iba1 in green) in the hippocampal section from vehicle mouse indicating the injection site (*). Scale bar = 200 μm. The dotted lines indicate the different areas of the hippocampal formation (CA1, CA2, CA3, and DG=Dentate Gyrus). **b**, **c**, **d** Histograms showing the number of Iba1^+^ cells by mm^2^ quantified in each area (CA1/CA2 in **b**, CA3 in **c**, and DG in **d**). Quantification of Iba1^+^ cells was analyzed from 3–5 sections per mouse (*n* = 3–4 for each group). In rmIL-33-injected mice, the number of microglia was exacerbated in all hippocampal areas. Interestingly, under minocycline treatment, these increases were drastically limited. Data are represented as mean ± SEM. Statistical comparisons were analyzed using Kruskal-Wallis and uncorrected Dunn’s test to generate P values for each paired comparison (each group vs. Sham without minocycline). *P ≤ 0.05

**Fig. 4:**
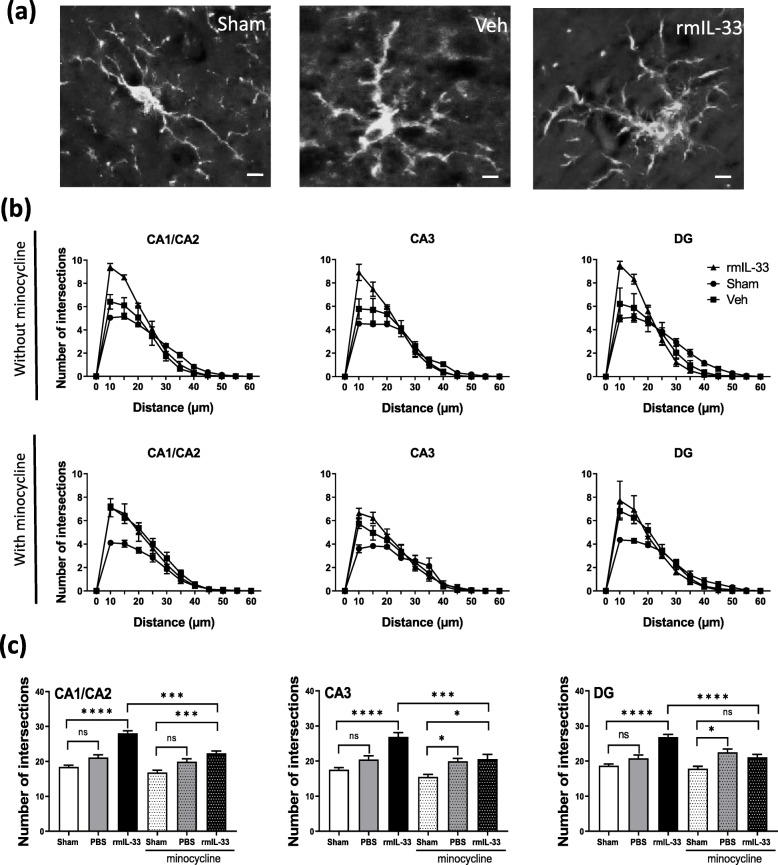
Effects of rmIL-33 treatment on microglial morphology in hippocampal areas. **a** Representative high magnification of Iba1^+^ microglia in sham, vehicle- or rmIL-33-treated mice (Scale bar = 10 μm). **b**, **c** Sholl analysis was performed after Iba1 immunohistochemistry on the hippocampal section from 48 h post-surgery mice. **b** To evaluate the ramification complexity of microglial cells, the number of process intersections per radius was reported graphically in the curve. **c** Bar graphs show the cumulated number of intersections at distances up to 25 μm from the soma. The analyses were performed for each hippocampal area and for each group without or with minocycline treatment. In all hippocampal areas, Sholl analysis showed that microglia maintained hyper-ramified state following IL-33 injection, although vehicle treatment induced an intermediate-ramified state, as compared to sham. The minocycline pre-treatment impaired IL-33 effect on microglial morphology. Data are represented as mean ± SEM. Statistical comparisons were made using One-way ANOVA followed by Tukey’s multiple comparison test. *P ≤ 0.05, ***P ≤ 0.001, ****P ≤ 0.0001

To further analyze IL-33 implication in the inflammatory processes, we next investigated the effect of rmIL-33 on microglia 48 h after intrahippocampal administration. Immunochemistry experiments were performed to quantify the number of Iba-1^+^ microglial cells in the different hippocampal areas (Fig. 3a). After vehicle injection, there was no significant difference in the number of Iba-1^+^ cells in the cornu ammonis (CA), CA1/CA2 (Fig. 3b), CA3 (Fig. 3c), and the dentate gyrus (DG) (Fig. 3d), as compared to sham groups. However, the number of Iba-1^+^ glial cells was increased after rmIL-33 administration in the three hippocampal areas. Interestingly, minocycline treatment prevented rmIL-33-induced increase of microglial cell numbers in all areas of the hippocampus. To go further, microglia activation was investigated at 48 h post-injection. Microglial cells exhibited a typical activated morphology 48 h after vehicle administration, as compared with sham controls and this activated phenotype was more prominent after rmIL-33 administration (Fig. 4a). The Sholl analysis was used to provide a quantitative assessment of glial cell activation in situ (Fig. 4b). We demonstrated an increase of proximal intersections per radius in the CA1/CA2, CA3, and the DG 48 h post-injection of vehicle, which was more pronounced after rmIL-33 treatment (Fig. 4b). Although hippocampal injection itself slightly modified microglia morphology, rmIL-33 significantly promoted the outgrowth of microglial processes, in agreement with an activated state. This effect of rmIL-33 administration was prevented by minocycline pre-treatment in the three hippocampal areas analyzed (Fig. 4b, c). These findings show that rmIL-33 administration induced microglial activation and proliferation/recruitment in the hippocampus, and this effect was sensitive to the anti-inflammatory effect of minocycline.

### Hippocampal exogenous rmIL-33 induces an increase of microglial cells expressing pro-IL-1β

**Fig. 5:**
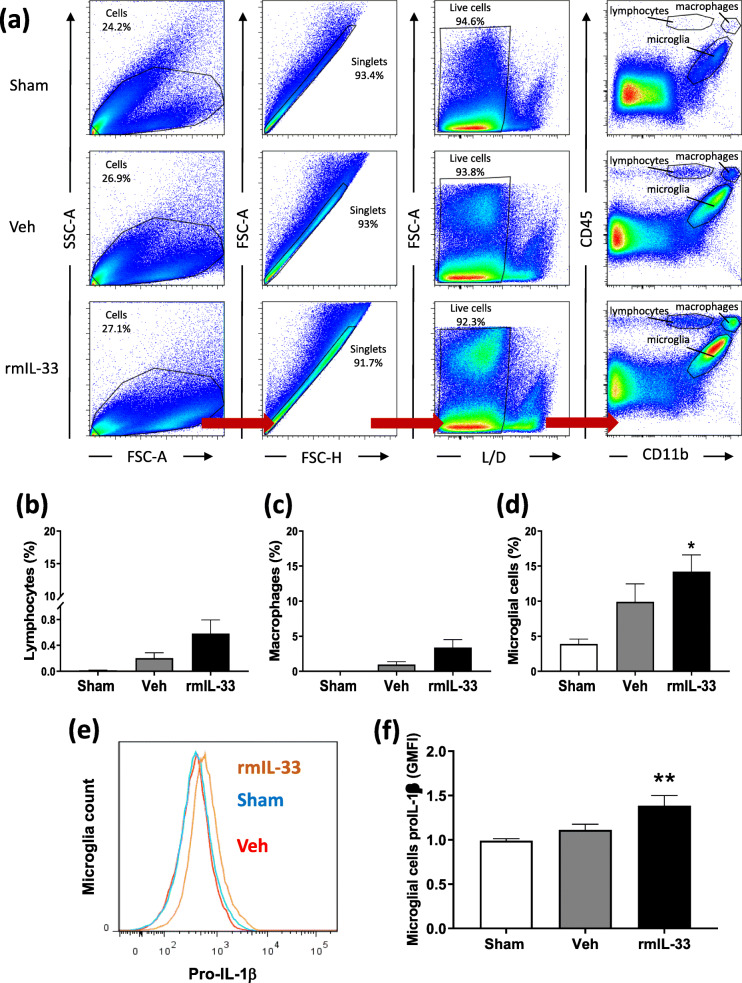
Increased IL-1β expressing microglia in the hippocampus of IL-33-treated mice. Flow cytometry revealing an increased proportion of microglia associated with a IL-1β pro-form overexpression. **a** Gating strategies to analyze whole hippocampus cell suspensions. A gate was created on non-debris population excluding very low SSC/FSC events. Then, cells were gated on single cells and selected on their live dead staining. This population was then gated according to CD11b and CD45 status as populations of CD11b^low^/CD45^high^ lymphocytes (**b**), CD11b^high^/CD45^high^ macrophages (**c**) and CD11b^high^/CD45^low^ microglia (**d**). The percentage of lymphocytes, macrophages, and microglia obtained from two experiments (*n* = 4–5 by the group) are graphically reported. **e** Representative geometric mean fluorescent intensity (GMFI) curve of IL-1β proform expression in microglial gated cells. **f** Microglial cells MFI for pro-IL-1β immunostaining expressed as ratio control relative to sham. Our flow cytometry analysis shows that compared with sham controls, rmIL-33 treatment induces a higher proportion of microglial cells correlated with a higher expression of IL-1β pro-form. Data are shown as mean ± SEM. Statistical analyses were made by Kruskal-Wallis test, followed by corrected Dunn’s multiple comparison test. *P ≤ 0.05, **P ≤ 0.01

To dissect the effect of rmIL-33 on microglia functions, we performed flow cytometry on dissociated cells from hippocampal tissues, 48 h after vehicle or rmIL-33 treatment. We determined the frequency of microglia, macrophages, and lymphocytes in hippocampal samples from sham, vehicle- and rmIL-33-treated groups. The gating strategy of live cell analysis is shown (Fig. 5a). Group comparison showed an increasing trend of CD11b^low^/CD45^high^ cells defined as lymphocytes and CD11b^+^/CD45^high^ cells defined as macrophages (Fig. 5b, c) after vehicle or rmIL-33 administration. However, there was an increase in terms of CD11b^+^/CD45^low^ cells defined as microglial cells in rmIL-33-treated mice, as compared with vehicle control group (Fig. 5d), in agreement with the immunohistochemical data (Fig. 3). Moreover, intracellular staining using a pro-IL-1β specific antibody demonstrated overexpression of pro-IL-1β by hippocampal microglial cells exposed to rmIL-33 (Fig. 5e, f). Altogether, these data suggest that rmIL-33 induced an increase of hippocampal microglial cells expressing pro-IL-1β.

### Exogenous rmIL-33-induced cognitive impairments require IL-1 signaling

As rmIL-33 administration induced microglia proliferation/recruitment with IL-1β overexpression, we next questioned whether IL-1 contributes to the maintenance of inflammation and the cognitive disorders induced by exogenous rmIL-33. To address this question, we injected rmIL-33 in the hippocampus of mice deficient for IL-1α and IL-1β (IL-1αβ^-/-^) and evaluated their responses in spatial memory tasks at 24 h and 48 h post-administration.

**Fig. 6:**
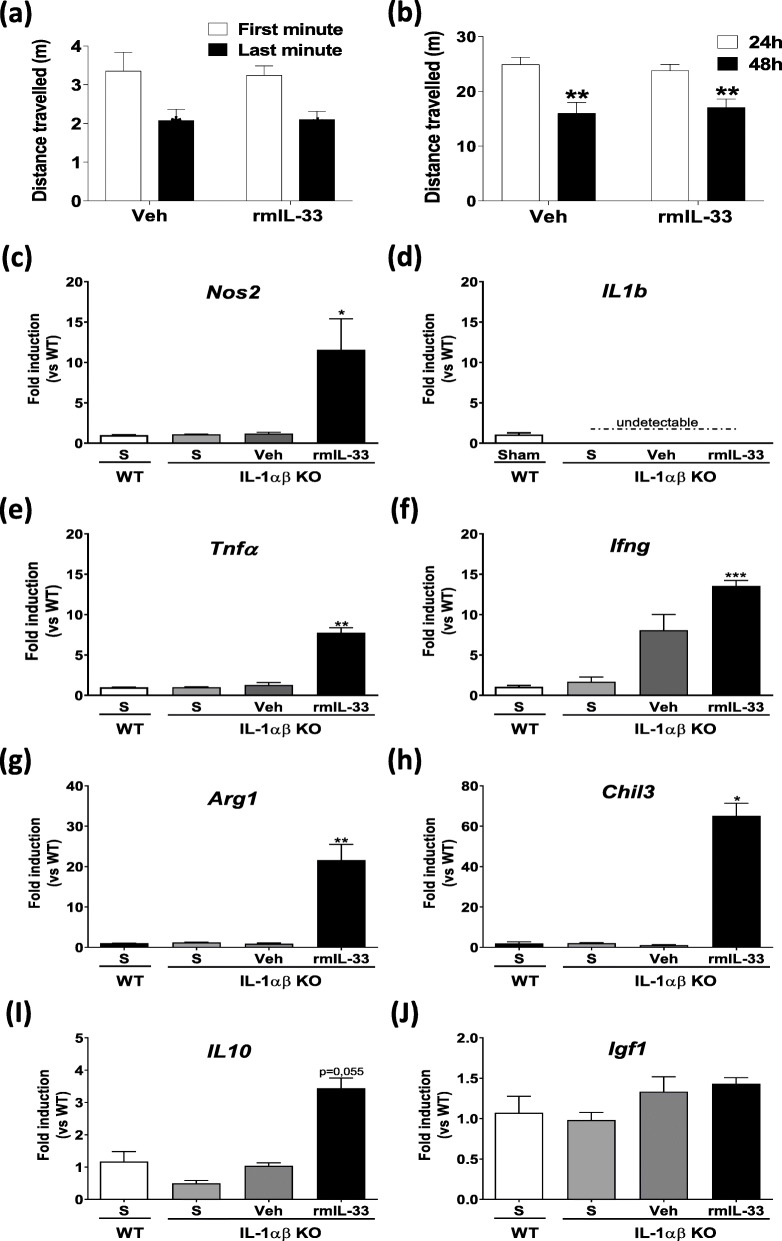
Effects of hippocampal exogenous IL-33 on spatial memory and habituation and hippocampi mRNA expression in IL-1αβ KO mice. IL-1αβ KO mice were intrahippocampally injected with rmIL-33 or vehicle solution (PBS-BSA 0.1%). Habituation in a novel environment was analyzed in intrasession (**a**) and in intersession 24 h and 48 h post-surgery (**b**). mRNA expression of pro-inflammatory markers (*Nos2* in **c**, *Il1b* in **d**, *Tnfa* in **e**, and *Ifng* in **f**) or anti-inflammatory markers (*Arg1* in **g**, *Chil3* in **h**, *Il10* in **i,** and *Igf1* in **j**) was quantified by RT-qPCR normalized against *18S* RNA at 48 h post-administration in WT/sham (WT-S), IL-1αβ KO/sham, IL-1αβ KO/vehicle and IL-1αβ KO/rmIL-33-treated groups (*n* = 4–6). Relative fold change in groups was quantified versus WT/sham group (S; white bar). Data are shown as mean ± SEM. The habituation analysis (**a**, **b**), *n* = 8–10 per group corresponding to a pool of 2 independent experiments. Two-way ANOVA followed by a Sidak post hoc test was used to analyze the distance traveled among the two groups (IL-1αβ KO injected with vehicle or rmIL-33). For qPCR analysis, statistical comparisons were made using Kruskal-Wallis followed by Dunn’s multiple comparison test for each group vs. WT/Sham. *P ≤ 0.05, **P ≤ 0.01, ***P ≤ 0.001

The decrease of distance traveled exhibited by vehicle-treated-IL-1αβ^-/-^ mice (Fig. 6a) was similar to WT mice at 24 h after rmIL-33 administration (Fig. 1b), indicating that IL-1αβ^-/-^ mice displayed as WT mice normal intrasession habituation. However, at 48 h post-rmIL-33 injection, the decrease in traveled distance indicating that IL-1αβ^-/-^ mice retained spatial memory retrieval (Fig. 6b), in contradiction with the rmIL-33-treated wild type mice, previously observed (Fig. 1c). Moreover, we showed that in the absence of IL-1αβ, rmIL-33 treatment induced an increase in the expression of key inflammatory mediators in the hippocampus (*Nos2, Tnfa, Ifng, Arg1, Chil3 and Il10*) of IL-1αβ^-/-^ mice, 48 h post-injection (Fig. 6c–j) which is similar in WT mice (Fig. 2). These results indicate that hippocampal IL-1 expression, and most likely IL-1β, is required for rmIL-33-induced cognitive impairment independently of upstream inflammatory mediators.

## Discussion

The implications of IL-33 has been described in many neuropathologies [1], not only as protective [14, 16] but also as disruptor [15, 17] of neuronal homeostasis. IL-33 exerts pleiotropic effects on the immune system, both on type 2 and type 1 immune responses, in the periphery but also at the CNS level. Despite the presence of IL-33 in a healthy brain [4] and in CNS pathologies [1], the multifold functions of IL-33 in CNS remain unclear. To elucidate the role of endogenous IL-33 in the CNS, the present study explored the consequences of intrahippocampal injection of recombinant IL-33 on cognitive function and neuroinflammatory processes.

Using spatial habituation tasks in an open field, allowing to address hippocampal non-associative learning and memory processes [19–21, 26], we show that the habituation to a novel environment was intact in IL-33 hippocampal treated mice 1-day post-surgery. These results indicate that neither the micro-lesion induced by the injection nor the IL-33 treatment had a neurological impact on learning at this stage. However, 48 h after intrahippocampal injection, IL-33-treated mice displayed a complete impairment of spatial memory retrieval. Unlike control mice, they were not able to recognize the previously explored environment, indicating that long-term habituation was significantly affected after rmIL-33 administration. These results suggest that a massive IL-33 release might disturb neuronal function and affect the memory retrieval process. IL-33 has been previously involved in cognitive defects observed in neuropathological conditions such as reflected in Alzheimer’s disease, multiple sclerosis, and experimental cerebral malaria [1, 10, 22]. Our data further show that injecting recombinant IL-33 directly in the hippocampus could mimic an acute exposure of IL-33 and its effects on cognitive processes.

To explore the link between the cognitive defect induced by IL-33 and the neuroinflammatory response, mice were pre-treated with minocycline. This antibiotic is able to cross the blood-brain barrier and exhibits anti-inflammatory properties preventing memory deficits in several neuropathologies [25]. In the present study, chronic administration of minocycline alone before intrahippocampal injections in control mice did not affect learning and spatial memory processes. However, our data also reveal that pre-treatment with minocycline seems to prevent the spatial memory retrieval impairment induced by IL-33 administration. This rescue of the IL-33-induced phenotype suggests that the cognitive impairments induced by IL-33 involved a neuroinflammatory process.

Previous studies demonstrated that IL-33/ST2 pathway modulated the production of cytokines and chemokines in neuropathological conditions [1, 3, 17]. We assessed the direct effects of IL-33 role on the inflammatory context by gene expression analysis. We quantified mRNA expression levels in the hippocampus of molecular markers usually used to define pro-inflammatory or regulatory immune response [27]. *Nos2*, *Il1b*, *Tnfa*, and *Ifng* are mediators of pro-inflammatory responses whereas *Arg1*, *Chil3*, *Il10,* and *Igf1* are associated with immunoregulatory mechanisms. In control mice, we observed a transient inflammatory response induced by the injection at 24 h and resolving at 48 h post-injection. This transient response to a slight trauma is correlated with the ability of the organism to return to a homeostasis state without adverse effects on behavior [28]. However, at 48 h, the intrahippocampal IL-33 injection induced a neuroinflammatory environment with overexpression of pro-inflammatory and immunoregulatory markers mRNA. Minocycline administration reduced this inflammatory context in terms of *Il1b* and *Ifng* expression at 48 h, contributing to the resolution of inflammation. These results suggest that exogenous IL-33 induces a neuroinflammatory phenotype associated with long-term habituation disturbance.

To explore the cellular process involved in IL-33-induced immune response, we focused on microglia, the first active immune barrier in the CNS strongly expressing IL-33 receptor ST2 [14]. We investigated the hippocampal microglia reaction by immunochemistry using Iba-1 staining. Indeed, in response to a neuroinflammatory context induced by LPS administration, resident microglia alter their shape in a specific way as compared with infiltrated peripheral cells with a rounder morphology [29, 30]. Sholl analysis on Iba1 immunofluorescent staining revealed a significant increase of proximal intersections per radius in the CA1/CA2, CA3, and DG regions 48 h after IL-33 treatment. This reactive morphology associated with an increase of microglial cell number demonstrated maintenance of their activated state. Minocycline administration through its anti-inflammatory activity attenuated the microglia activation of IL-33 treated mice, in line with previous reports in cognitive disorders [31, 32]. This result suggests that the deleterious function of IL-33 pathway on spatial memory retrieval processes requires microglia activation, especially in the hippocampal formation. Indeed, in healthy conditions, microglia regulate neuronal activity, synaptic plasticity, and adult neurogenesis required for learning and memory. In many neuropathologies, the microglia reactivity state has been characterized based on morphological modifications and the release of cytokines, chemokines, and growth factors, modulating neuronal and synaptic functions. This activated phenotype should be beneficial and associated with inflammatory changes to combat the injury and return to a homeostatic state. However, these defense processes could be over-stimulated and cause significant damage to behavior [33], as we demonstrated hereafter intrahippocampal IL-33 injection.

To confirm the IL-33-induced microglial reactivity, cells from the hippocampi of IL-33-treated mice were analyzed by flow cytometry at 48 h post-surgery. Although neither macrophage nor lymphocyte recruitment was observed, microglia number was significantly increased in IL-33-treated mice, confirming our immunohistological data. This analysis revealed also an overexpression of the IL-1β immature form in hippocampal microglia 48 h after IL-33 injection. Thus, in vivo, IL-33 treatment promotes IL-1β microglia production, as previously demonstrated in vitro [10], indicating that microglia contribute to the pro-inflammatory response. We then hypothesized that the cognitive impairment induced by exogenous IL-33 may be mediated in part by microglia derived IL-1. To test this point, we performed IL-33 intrahippocampal microinjections in IL-1αβ deficient mice. The absence of IL-1 cytokines prevented spatial memory retrieval impairment induced by IL-33 administration even if neuroinflammatory markers, except IL-1β and IL-1α, were upregulated. Thus, IL-1β-producing microglia are required for IL-33 neurotoxic effects on cognitive impairment. In our experimental conditions, IL-1 contribution to these cognitive defects impairment could involve its non-immunological activities. Indeed, this cytokine has been described as critical for learning and memory in a dose-dependent manner [34]. Here, we verified that IL-1αβ deficient mice behave as WT mice in terms of habituation to spatial novelty in our experimental conditions. Prolonged up-regulation of pro-inflammatory cytokines, especially IL-1β, has been associated with a decrease in synaptic plasticity, as well as a deficit in spatial learning [35, 36]. This IL-1β disruptive effect on cognitive functions could involve the inhibition of long-term potentiation generation at the neuronal level and/or defect of neurotrophic factors production [34]. All these parameters should be further investigated in our futures studies.

## Conclusion

In conclusion, we showed that IL-33 intrahippocampal administration is a valuable tool to mimic a local acute exposition. We provide the evidence that CNS IL-33 directly orchestrates neuroinflammatory mechanisms through microglia activation and overproduction of IL-1-inducing spatial memory disorders. Thus, we suggest that in neuropathological conditions IL-33 released by astrocytes and/or oligodendrocytes may activate microglia and induce IL-1-dependent cognitive defects. These results highlight the need to dissociate the CNS versus systemic IL-33 effects, in particular in the context of cerebral diseases.

## Supplementary information

**Additional file 1.** The emotional state of mice was tested 24H after the intrahippocampal microinjection. Anxiety-like behavior, representing by the time spent in center of the open field, was not different between: (a) mice intrahippocampal injected with vehicle solution (Veh) versus rmIL-33, (b) mice intrahippocampal injected with vehicle solution versus rmIL-33 treated with minocycline, (c) IL-1αβ KO mice intrahippocampal injected with vehicle solution versus rmIL-33. Values are mean ± SEM, n = 8-15 per group, Mann-Whitney test was used to compare the time spent in the center between 2 groups.

**Additional file 2.** Expression of IL-1a mRNA in hippocampi at 24 h and 48 h post-surgery, in Sham, vehicle or IL-33 intra-hippocampal treated mice with or without minocycline pretreatment. mRNA expression of the pro-inflammatory marker IL-1a was quantified in hippocampi by RT-qPCR normalized against 18S RNA. Relative fold change in vehicle group (grey bar) and in IL-33 group (black bar) were quantified versus sham group (S ; white bar). Minocycline treated mice were also analyzed at 48 h post-surgery (dotted bar). IL-33 injection delayed the resolution of inflammation highlighted by an increase of this markers at 48 h administration. A partial reduction of this effect was observed under minocycline exposure. Data are represented as mean ± SEM (n = 4-6). Statistical comparisons were made using Kruskal-Wallis followed by Dunn’s multiple comparison test for each group vs. Sham.**p ≤ 0.01, ***p ≤ 0.001. In addition, comparison between rmIL-33 (48 h) and rmIL-33 + mino (48 h) groups was performed using the Mann-Whithney test.

## Data Availability

The datasets used and/or analyzed during the current study are available from the corresponding author on reasonable request.
