# Supplementary figures and images for: Hippocampal interleukin-33 mediates neuroinflammation-induced cognitive impairments
Source: J Neuroinflammation. 2020 Sep 11;17:268. doi: 10.1186/s12974-020-01939-6 (PMC7488545; doi:10.1186/s12974-020-01939-6)

Addition file 1

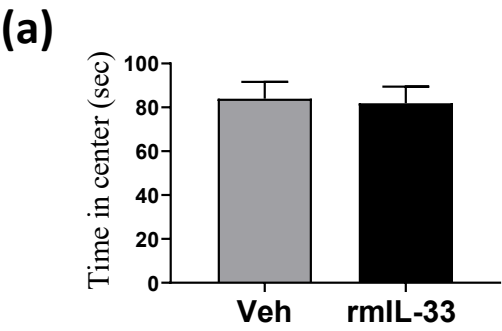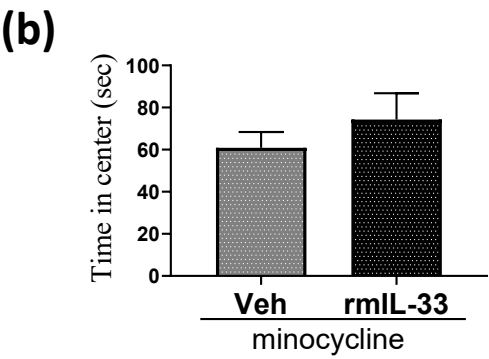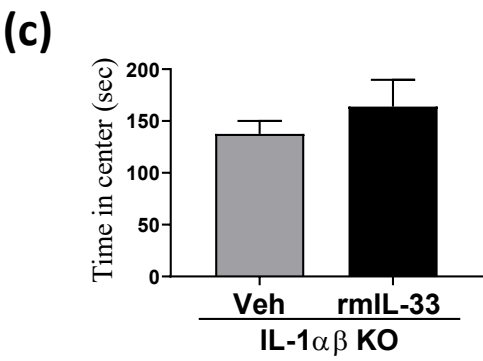

Supplement: Supplementary file 1 — Additional file 1. The emotional state of mice was tested 24H after the intrahippocampal microinjection. Anxiety-like behavior, representing by the time spent in center of the open field, was not different between: (a) mice intrahippocampal injected with vehicle solution (Veh) versus rmIL-33, (b) mice intrahippocampal injected with vehicle solution versus rmIL-33 treated with minocycline, (c) IL-1αβ KO mice intrahippocampal injected with vehicle solution versus rmIL-33. Values are mean ± SEM, n = 8-15 per group, Mann-Whitney test was used to compare the time spent in the center between 2 groups. [file 12974_2020_1939_MOESM1_ESM.pdf]

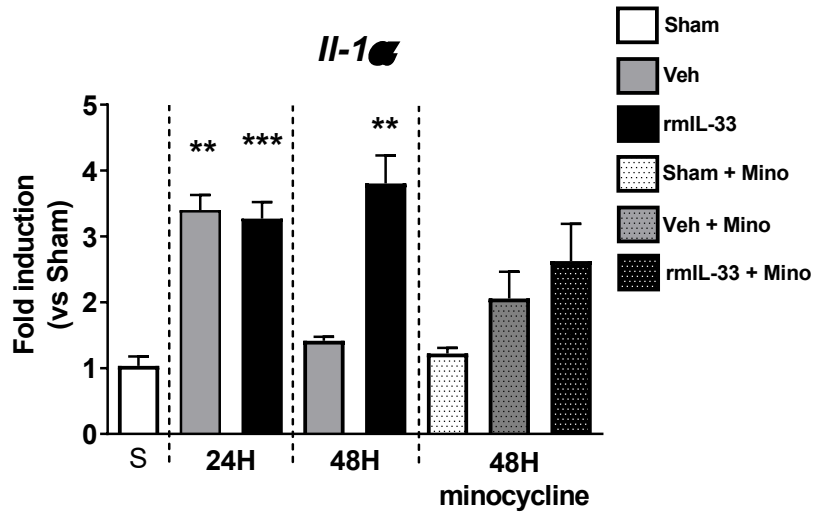

Supplement: Supplementary file 2 — Additional file 2. Expression of IL-1a mRNA in hippocampi at 24 h and 48 h post-surgery, in Sham, vehicle or IL-33 intra-hippocampal treated mice with or without minocycline pretreatment. mRNA expression of the pro-inflammatory marker IL-1a was quantified in hippocampi by RT-qPCR normalized against 18S RNA. Relative fold change in vehicle group (grey bar) and in IL-33 group (black bar) were quantified versus sham group (S ; white bar). Minocycline treated mice were also analyzed at 48 h post-surgery (dotted bar). IL-33 injection delayed the resolution of inflammation highlighted by an increase of this markers at 48 h administration. A partial reduction of this effect was observed under minocycline exposure. Data are represented as mean ± SEM (n = 4-6). Statistical comparisons were made using Kruskal-Wallis followed by Dunn’s multiple comparison test for each group vs. Sham.**p ≤ 0.01, ***p ≤ 0.001. In addition, comparison between rmIL-33 (48 h) and rmIL-33 + mino (48 h) groups was performed using the Mann-Whithney test. [file 12974_2020_1939_MOESM2_ESM.pdf]
